# BAP31 Inhibits Cell Adaptation to ER Stress Conditions, Negatively Regulating Autophagy Induction by Interaction with STX17

**DOI:** 10.3390/cells8111350

**Published:** 2019-10-30

**Authors:** Kayo Machihara, Takushi Namba

**Affiliations:** 1 Research and Education Faculty, Multidisciplinary Science Cluster, Interdisciplinary Science Unit, Kochi University, Kohasu Oko-cho Nankoku-shi, Kochi 783-8505, Japan; 2 Graduate School of Medicine, Kochi University, Nankoku 783-8502, Japan; 3 Department of Marine Resource Science, Faculty of Agriculture and Marine Science, Kochi University, Nankoku 783-8502, Japan

**Keywords:** ER stress, autophagy, BAP31, STX17, tumor suppression, stress adaptation

## Abstract

Cancer cells modulate their metabolism to proliferate and survive under the metabolic stress condition, which is known as endoplasmic reticulum (ER) stress. Therefore, cancer cells should suppress ER stress-mediated cell death and induce autophagy—which recycles metabolites to provide energy and new macromolecules. In this study, we demonstrate that the ER membrane protein BAP31 acts to suppress adaptation to ER stress conditions, induce cell death, and suppress autophagy by forming a BAP31-STX17 protein complex. The loss of BAP31 stimulates tumor growth in metabolic stress conditions in vivo and enhances invasion activity. Therefore, BAP31 stimulates cell death and inhibits autophagy, and it can be considered a novel tumor suppressor factor that acts by preventing ER stress adaptation.

## 1. Introduction

To survive and proliferate under metabolic stress, which restricts the availability of essential nutrients (e.g., oxygen, amino acids, and glucose), due to unregulated tumor growth and uncontrolled angiogenesis, cancer cells show distinct aberrations in their metabolic pathways, including protein synthesis and glucose metabolism [1,2,3]. Under metabolic stress, cancer cells need to synthesize large amounts of protein because of their rapid growth, produce nutrients and induce cellular reactions, such as autophagy [4]. The endoplasmic reticulum (ER) is a dynamic organelle that is required for the proper localization, modification, and folding of proteins. Metabolic stress induces ER dysfunction due to accumulation of unfolded proteins in the ER, inhibited protein glycosylation, and lack of essential nutrients, and then also induces the unfolding protein response (UPR). UPR maintains and restores ER homeostasis to increase protein secretion by inducing the ER chaperons, such as binding immunoglobulin protein (BiP), that mediate protein refolding and degrade unfolded proteins by ER-associated degradation [5]. However, unrecoverable ER stress induces cell death to eliminate damaged cells [6]. Autophagy can play a crucial role in cellular adaptation to starvation. By recycling metabolites, autophagy provides a source of energy and basic factors for the biosynthesis of new macromolecules [7]. Therefore, resistance to ER stress and the induction of autophagy is essential for tumor proliferation and survival, and the suppression of these processes is an important “metabolic checkpoint” for the elimination of cancerous cells; however, the molecular mechanism by which cancerous cells avoid the metabolic checkpoint is not fully understood.

B-cell receptor associated protein 31 (BAP31) is a ER membrane protein that functions in a number of apoptotic pathways involving the cleavage of BAP31 by caspase-8 [8,9], and BAP31 functions as a regulator of cell death through interaction with Bcl-2 or Bcl-XL and caspase-8 [10,11]. Recently, we discovered that BAP31 regulates mitochondrial function and metabolic pathways, such as autophagy, by communication with ER and mitochondria via contact sites [12]; however, the role of BAP31 in the metabolic checkpoint and cancerous phenotype is not fully understood.

This study reports that BAP31 inhibits resistance to metabolic stress in cancer cells, induces cell death via ER stress, and acts as a tumor suppressor factor by suppressing autophagy via the metabolic checkpoint. BAP31 interacts with syntaxin 17 (STX17), which suppressed the induction of autophagy by suppressing interaction of STX17 with autophagy related protein 14L (ATG14L). Moreover, BAP31-knockout enhances invasion activity and tumor growth in metabolic stress conditions in vivo compared to BAP31 expressing cells. Therefore, BAP31 can be considered a novel tumor suppressor factor involved with metabolic stress, inducing cell death via the ER stress response and suppressing autophagy by inhibiting STX17 and ATG14L interaction.

## 2. Materials and Methods

### 2.1. Cell Lines and Generation of Stable Cell Lines

The U2OS and HeLa cells were cultured in Dulbecco’s Modified Eagle’s Medium (DMEM) supplemented with 10% fetal bovine serum (FBS), 100 units/mL penicillin and 100 µg/mL streptomycin. The mouse embryonic fibroblast (MEF) cells were cultured in DMEM supplemented with 10% FBS, 1× NEAA and 0.1% β-mercaptoethanol. NB1RGB cells were cultured in α-MEN supplemented with 10% FBS, 100 units /mL penicillin and 100 µg/mL streptomycin. All cells were cultured at 37 °C in 5% CO_2_. MEF cells were obtained from Dr. Lee (Harvard University, Boston, MA, USA). U2OS and HeLa cell lines were obtained from ATCC (Manassas, VA, USA). NB1RGB cells were obtained from Riken cell bank (Tsukuba, Japan).

To generate stable cell lines expressing GFP-LC3 (pEGFP-LC3; Addgene #21073, Cambridge, MA, USA) or transiently expressed Flag-STX17 (Addgene #45911, Cambridge, MA, USA), pcDNA3.1 (control) (invitrogen-Thermo Fisher Scientific, Hampton, NH, USA), HA-BAP31 [13], HA-ATG14L (Addgene #24294, Cambridge, MA, USA) or DsRed-ER (Takara Bio Inc, Tokyo, Japan) constructs were introduced into the U2OS cells using lipofection methods. The plasmids (GFP-LC3) were transfected into the U2OS cells and selected with G418 for two weeks. The experiments were performed using single clones (GFP-LC3).

To generate the BAP31 knockout cells, the U2OS cells were transfected with a clustered regularly interspaced short palindromic repeat (CRISPR) ribonucleoprotein complex containing Alt-R CRISPR target crRNA or negative control crRNA:tracrRNA and Cas9 nuclease according to the manufacturer’s protocol (Integrated DNA Technologies, Coraville, IA, USA). To target human BAP31, the following sgBAP31 sequence was used: sgBAP31-2; 5′-TGCCACCTTCCTCTATGCGG-3′, sgBAP31-3; 5-GTGAACCTCCAGAACAATCC-3.

### 2.2. Immunoblotting Analysis

Immunoblotting experiments were conducted as previously described [13]. Antibodies used for immunoblotting were specific for the proteins as follows: LC3b, BiP, PARP, Beclin 1, ATG14L, ATG12, p62 and ATG7 (Cell Signaling, Beverly, MA, USA); BAP31 (Santa Cruz, Santa Cruz, CA, USA); and STX17, β-actin, and HA (Sigma, St. Louis, MO, USA). Antibodies were diluted to 1:1000, or anti-β-actin (1:10000). Secondary antibodies were purchased from Promega (Madison, WI, USA) (anti-rabbit and anti-mouse at 1:5000).

### 2.3. Xenograft Model

The Animal Research Committee of Kochi Medical School approved all experimental protocols and surgical procedures (Permit Number: K-00010), which were performed according to the guidelines for proper conduct of animal experiments from the Science Council of Japan. All studies involving animals are reported according to the ARRIVE guidelines for reporting experiments involving animals. Each BALB/c nude mouse (Crea-Japan Inc. Tokyo, Japna) (Male; 5 weeks of age) was subcutaneously inoculated in the right and left hind footpads with 5 × 10^6^ U2OS sgNegative, U2OS sgBAP31-2 or U2OS sgBAP31-3 cells. Four days after the inoculation, the tumors were measured every 7 days, and their volumes were calculated using the following equation: mm^3^ = [length (mm)] × [width (mm)]^2^/2).

### 2.4. Immunoprecipitation

Immunoprecipitation experiments were conducted as previously described [12]. Briefly, the cells were incubated with PBS containing 1 mM dithiobis [succinimidyl propionate] (DSP) for 30 min, and then, the reaction was quenched by 50 mM Tris (pH 8.0) for 3 min. The cells were lysed and incubated on ice for 10 min. The cellular debris were pelleted by centrifugation. The primary antibody was covalently immobilized onto protein A/G plus-agarose, followed by immunoprecipitation steps using a Crosslink Immunoprecipitation Kit (Pierce-Thermo Fisher Scientific, Hampton, NH, USA) according to the manufacturer’s protocol. The immunoprecipitated products were incubated with a LDS sample buffer containing 50 mM DTT at 95 °C for 10 min.

### 2.5. Immunostaining

The cells were cultured on poly-L-lysine coated coverslips and fixed in 4% formaldehyde and permeabilized in 0.1% Triton X-100. The primary antibodies included an anti-STX17 polyclonal, anti-FLAG polyclonal (Sigma), anti-BAP31 monoclonal (Santa Cruz, Santa Cruz, CA, USA) and anti-ATG14L monoclonal (MBL Lifescience, Tokyo, Japan) antibodies. The secondary antibodies were fluorescein isothiocyanate (FITC)-conjugated for rabbit IgG and Rhodamine Red or FITC-conjugated for mouse Immunoglobulin G (IgG) (Molecular Probes, Hampton, NH, USA). The DAPI stained nuclei (Roche, Indianapolis, IN, USA). An in situ proximity ligation assay (PLA) and immunofluorescence were performed to detect the BAP31 and STX17 or STX17 and ATG14L interactions using primary antibodies and a Duolink II Detection Kit with PLA PLUS and MINUS Probes for rabbit and mouse according to the manufacturer’s protocol (Olink Bioscience, Uppsala, Sweden). All images were obtained under a confocal microscope (Olympus, Tokyo, Japan) and processed using Adobe Photoshop software (CS6; Adobe, San Jose, CA, USA).

### 2.6. siRNA Targeting of Genes

The U2OS and MEF cells were transfected with small interfering RNA (siRNA) for BAP31 [14] (the Smart Pools of siRNAs for human BAP31, ATG7 or mouse BAP31 from Dharmacon, Lafayette, CO, USA), STX17 (5′-GGAAACCTTAGAAGCGGACTTAATT-3′ Stealth RNAi oligonucleocide, Invitrogen, Carlsbad, CA, USA) and Control (Santa Cruz, Santa Cruz, CA, USA) using Lipofectamine RNAimax transfection reagent (Invitrogen, Carlsbad, CA, USA) according to the manufacturer’s instructions.

### 2.7. Cell Viability Assay

Cell viability was determined using the 3-(4,5-di-methylthiazol-2-yl)-2,5-diphenyltetrazolium bromide (MTT) method. Cells were treated with indicated methods and incubated with MTT solution (1 mg/mL) for 1 to 2 h. Isopropanol/HCl were then added to final concentrations of 50% and 20 mM, respectively, and measured absorbance at 570 nm was determined using an infinite F50R spectrophotometer (TECAN, Tokyo, Japan).

### 2.8. Annexin V Binding by FACS

Detection of early phase of apoptosis was done using the Annexin V-FITC kit (MBL, Tokyo, Japan). According to the manufacturer’s protocols, cells were washed with a culture medium and PBS, and then re-suspended in the binding buffer. After the addition of Annexin V-FITC and PI solutions, samples were incubated for 15 min at room temperature (RT) in the dark. Samples were scanned with a BD LSRFortessa X-20 and analyzed by FlowJo software. Plots in the Annexin V-FITC-positive (+)/PI-negative (−) quadrant were counted as apoptotic cells.

### 2.9. Cell Invasion Assay

The invasion assay was performed using a BioCoat Matrigel invasion chamber system (Corning, Corning, NY, USA) according to the manufacturer’s protocol with certain modifications [15]. Briefly, the Transwell chamber was filled with a culture medium containing 10% FBS and a cell suspension containing 1% FBS was applied to the Matrigel-coated upper chamber. The plate was incubated at 37 °C for 24 h. The cells were removed from the upper surface of the membrane, and the lower surface of the membrane was stained for 10 min with 1% crystal violet in 25% methanol, rinsed with distilled water, and air-dried. The crystal violet was then extracted and the absorbance was measured at 570 nm.

### 2.10. Statistical Analysis

The differences in the mean values were evaluated using two-way ANOVA, followed by Tukey’s test. The differences were considered significant at *P* < 0.05.

## 3. Results

### 3.1. ER Stress Induces Autophagy, which Antagonizes Cell Death

**Figure 1:**
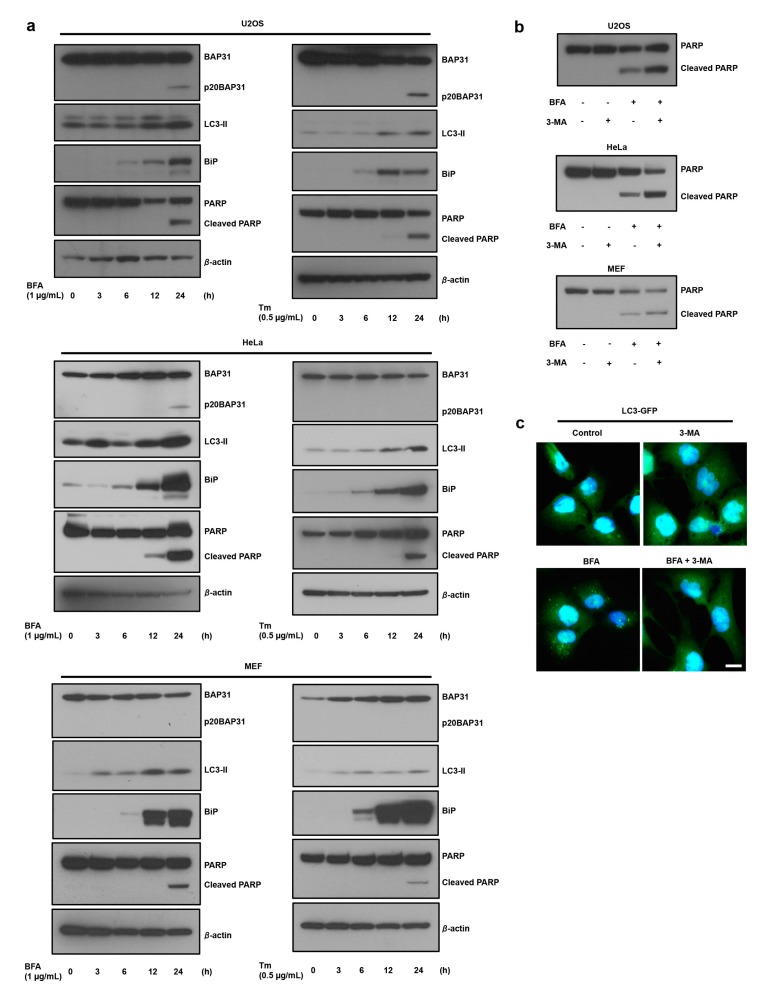
ER stress induces autophagy, which suppressed ER stress-induced cell death. (**a**) ER stress induces cell death and autophagy. U2OS, HeLa, and MEF cells were treated with the indicated compounds at the indicated concentrations for the indicated time. Cell lysates were subjected to immunoblotting using anti-BAP31, anti-LC3, anti-BiP, anti-PARP, and anti-β-actin antibodies. Three independent experiments were done and quantification analysis is shown in Figure S1a. (**b**) The suppression of the induction of autophagy stimulates ER stress-induced cell death. U2OS, Hela, and MEF cells were preincubated with 5 mM of 3-MA for 1 h and further incubated with or without brefeldin A (BFA) (1 µg/mL) for 18 h. Cell lysates were subjected to immunoblotting using anti-PARP antibody. Three independent experiments were done and quantification analysis is shown in Figure S1b. (**c**) U2OS cells stably expressing GFP-LC3 were preincubated with 5 mM of 3-MA for 1 h and further incubated with or without BFA (1 µg/mL) for 18 h. Cells were fixed with 4% paraformaldehyde (PFA), and GFP-LC3 (green) fluorescence was determined. Blue represents nuclear 4’,6-diamidino-2-phenylindole (DAPI) staining. Scale bar, 10 μm.

Numerous reports have suggested that ER stress-induced autophagy is important to the adaptation of ER stress conditions [16]. We first confirmed the role of autophagy in the ER stress response. As expected, the levels of the autophagy marker LC3-II increased in response to ER-specific stress (brefeldin A, BFA; and tunicamycin, Tm), and this induction occurred earlier than the cell death-mediated PARP cleavages in U2OS, HeLa, and MEF cells (Figure 1a) and quantification data was shown in Figure S1a. During cell death, caspase-8 causes the cleavage of BAP31 into a p20BAP31 fragment that is known to function as a pro-apoptotic factor [17]. The generation of the pro-apoptotic p20BAP31 fragment was dependent on the cell type and treatment agent (Figure 1a). In addition, 3-methyladenine (3-MA)-induced inhibition of autophagy significantly stimulated ER stress-induced PARP cleavage in U2OS, HeLa, and MEF cells (Figure 1b and Figure S1b). 3-MA also suppressed ER stress-induced LC3-GFP puncta (Figure 1c). Using *ATG7* knockdown, we determined whether a different autophagy inhibition method stimulates ER stress-induced cell death. Figure S2a shows that *ATG7* knockdown suppressed ER stress-induced autophagy and significantly stimulated Poly (ADP-ribose) polymerase (PARP) cleavage in U2OS cells (Figure S2b). These results indicate that autophagy has a protective role in ER stress-induced cell death.

### 3.2. The Loss of BAP31-Suppressed ER Stress-Induced Cell Death by Inducing Autophagy

**Figure 2:**
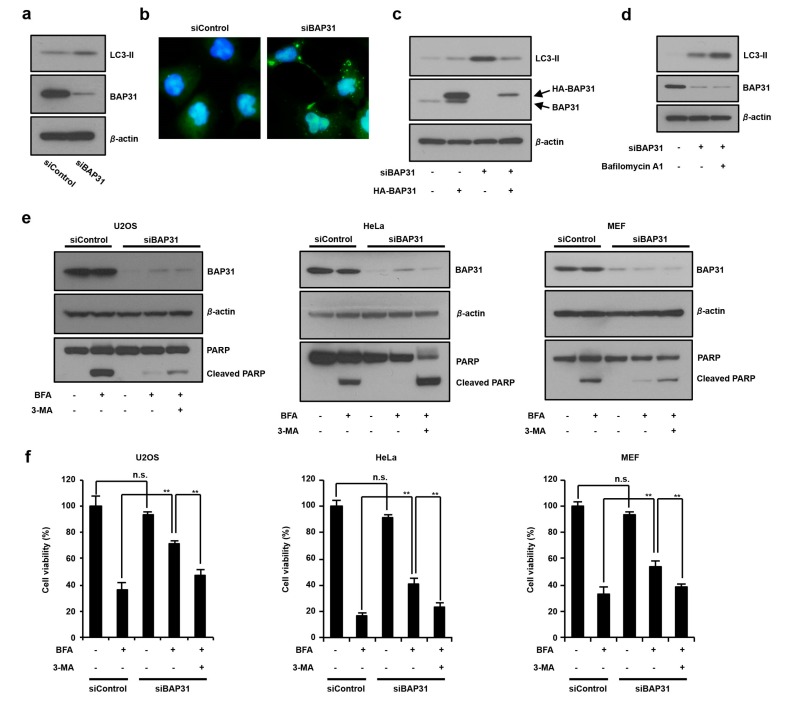
The suppression of BAP31 expression induces autophagy and antagonizes ER stress-induced cell death. (**a**) Loss of BAP31 increases LC3-II expression. U2OS cells were transfected with 150 pmol of siBAP31 or siControl for 24 h. Cells were subjected to immunoblotting using anti-BAP31, anti-LC3, and anti-β-actin antibodies. (**b**) U2OS cells stably expressing GFP-LC3 were transfected with 150 pmol of siBAP31 or siControl for 24 h. Cells were fixed with 4% PFA, and GFP-LC3 (green) fluorescence was determined. Blue represents nuclear DAPI staining. Scale bar, 10 μm. (**c**) U2OS cells were transfected with siBAP31 (+) or siControl (−) for 18 h and then transfected with HA-BAP31 (+) or pcDNA3.1 (−) for 12 h. Cells were subjected to immunoblotting using indicated antibodies. (**d**) *BAP31* knockdown stimulates autophagosome synthesis. U2OS cells were transfected with 150 pmol of siBAP31 or siControl for 24 h, followed by treatment with or without 1 µg/mL of bafilomycin A1 for 1 h. Cells were subjected to immunoblotting using the indicated antibodies. (**e**,**f**) The suppression of BAP31 inhibits ER stress-mediated cell death by inducting autophagy. The cells were transfected with siBAP31 or siControl for 16 h and these cells were preincubated with or without 5 mM of 3-MA for 1 h and further incubated with or without BFA (1 µg/mL) for 18 h in U2OS, HeLa, and MEF cells. Cells were subjected to immunoblotting using the indicated antibodies (**e**) or MTT using cell viability assay (**f**). These experiments were repeated two times (**a**–**e**). Data are presented as the mean ± standard deviation (SD) of the three simultaneously performed experiments (**f**). *P* values were calculated using two-way ANOVA; n.s., not significant; ** *P* < 0.01 (**f**).

We reported that loss of BAP31 increased autophagy via activation of AMP-activated protein kinase (AMPK) signaling [12]. In this study, we tested the role of autophagy in the BAP31 knockdown-mediated suppression of ER stress-induced cell death. U2OS cells were treated with siRNA to *BAP31* to suppress expression of the BAP31 protein, and autophagy marker LC3-II levels were monitored. As shown in Figure 2a,b, *BAP31* knockdown by siRNA silencing increased LC3-II protein expression and LC3-GFP puncta. To exclude the possible off-target effects of siRNA on BAP31, we examined the effect of re-expression of BAP31. We observed that *BAP31* knockdown increases LC3-II expression. This increased LC3-II expression suppressed HA-BAP31 re-expression in siBAP31-treated cells (Figure 2c). In addition, HA-BAP31 overexpression suppressed ER stress-induced autophagy (Figure S3). We investigated whether *BAP31* knockdown increases LC3-II expression due to enhanced autophagosome formation or blockage of autophagosome–lysosome fusion. Increased LC3-II expression provides evidence of efficient autophagic flux in the presence of bafilomycin A1, which inhibits autolysosome degradation. As shown in Figure 2d, siBAP31 and bafilomycin A1 cotreatment stimulated LC3-II expression compared to siBAP31 treatment. We also confirmed that *BAP31* knockdown reduced p62 protein expression levels, suggesting that *BAP31* knockdown induces autophagosome synthesis (Figure S4). These results suggested that BAP31 suppresses autophagy induction.

*BAP31* knockdown cells suppressed the ER stress-induced PARP cleavage and increased cell viability compared to siControl- and BFA-treated cells, and additional treatment with the autophagy inhibitor stimulated PARP cleavage and suppressed cell viability in U2OS, HeLa, and MEF cells (Figure 2e,f). We also observed that ER stress-induced early apoptosis was affected by *BAP31* knockdown–inhibited induction of autophagy using Annexin V-FITC and propidium iodide (PI) staining. As shown in Figure S5a,b, *BAP31* knockdown slightly increased apoptosis of annexin V-positive and PI-negative cells, although not significantly, but suppressed ER stress-induced apoptosis. Additional treatment with 3-methyladenine (3-MA) stimulated apoptosis in U2OS and HeLa cells. These results indicated that BAP31 regulates autophagy induction and that of BAP31 suppression inhibits ER stress-induced cell death by inducing autophagy.

### 3.3. Identification of STX17 as a BAP31 Binding Partner and How This Complex Regulates Induction of Autophagy via Suppression of STX17 and ATG14 Interaction

**Figure 3:**
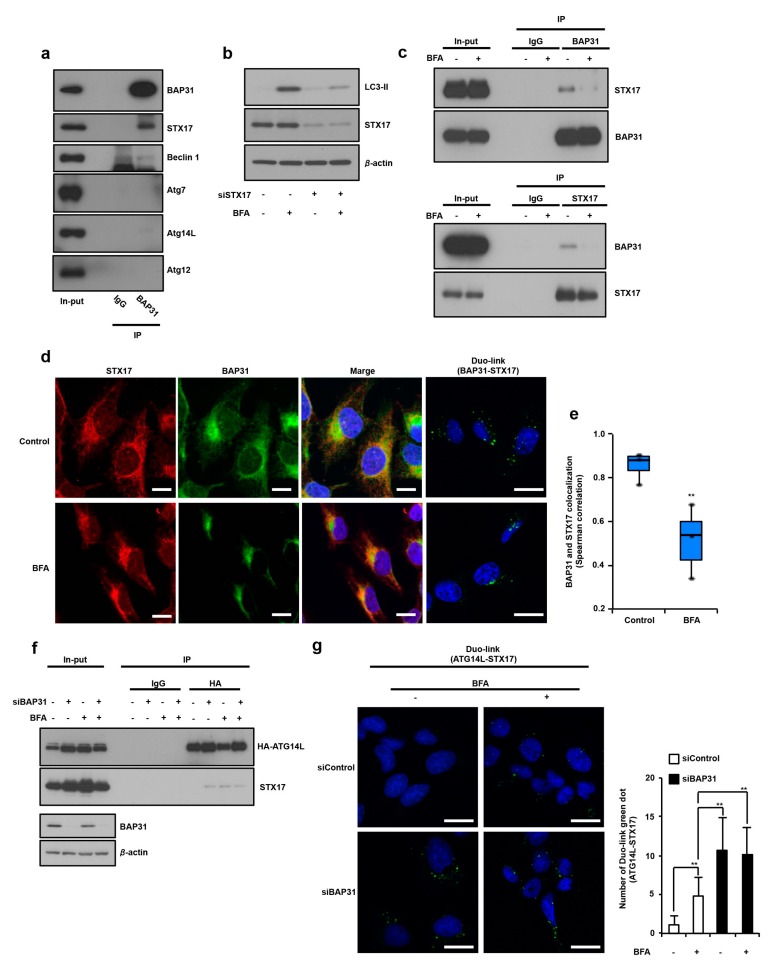
BAP31 interacts with STX17, and disintegration of this complex stimulates STX17 and ATG14L interaction by through BAP31 deficiency. (**a**) U2OS cells were harvested, and the proteins were cross-linked with DSP prior to the protein extraction. A co-immunoprecipitation assay was performed with cell lysates, followed by western blotting, using the indicated antibodies. (**b**) *STX17* knockdown suppressed ER stress-induced autophagy. U2OS cells were transfected with siControl (−) or siBAP31 (+) for 24 h and these cells were then treated with (−) or without (+) BFA (1 µg/mL) for 18 h. Cells were subjected to immunoblotting using indicated antibodies. (**c**) BAP31 and STX17 interaction is suppressed by ER stress. U2OS cells were treated with or without BFA (1 µg/mL) for 12 h. Co-immunoprecipitation was performed using the same procedure described in Figure 3a. These experiments were repeated two times (**a**–**c**). (**d**) U2OS cells were treated with BFA (1 µg/mL) for 12 h and subjected to immunostaining analysis using antibodies to BAP31 (green) and STX17 (red). Merged images are also shown, and co-localization is indicated in yellow. Blue represents nuclear DAPI staining (scale bar, 10 µm). Representative images of DuoLink using BAP31 and STX17 (right panel) antibodies in U2OS cell. (**e**) Co-localization analysis using the Fiji Coloc 2 (Spearman correlation) was conducted to examine pixel intensity correlations over space in single confocal image. Graphs show medians, interquartile ranges, and min/max values. The seven simultaneously performed experiments. (**f**) U2OS cells were transfected with (+) or without (−) HA-ATG14 and siControl (−) or siBAP31 (+) for 24 h and these cells were then treated with (−) or without (+) BFA (1 µg/mL) for 8 h. A co-immunoprecipitation assay was performed with cell lysates, followed by western blotting, using the indicated antibodies. This experiment was repeated two times (**f**). (**g**) The endogenous ATG14L and STX17 interactions detected by performing a proximity ligation assay are shown as green dots. U2OS cells were transfected with siControl (−) or siBAP31 (+) for 24 h and these cells were treated with (−) or without (+) BFA (1 µg/mL) for 8 h. Blue represents nuclear DAPI staining (scale bar, 10 µm). The number of DuoLink dots (BAP31-ATG14L interaction) in the cells was determined (*n* = 6) (**d**, right panel). Data are presented as the mean ± standard deviation (SD). The *P*-value was calculated using two-way ANOVA. ** *P* < 0.01 (**d**).

The autophagy related protein (ATG) family and the ER-resident SNARE protein syntaxin 17 (STX17) are well-known factors in the induction of autophagy signaling [18,19]. However, whether BAP31 interacts with these autophagy-induced factors is not clear. Therefore, we aimed to understand the role of BAP31 in autophagy signaling and to identify possible BAP31-binding autophagy-induced factors, including STX17, Beclin1 (ATG8), ATG7, ATG12, and ATG14L. We first analyzed the interaction between BAP31 and these above proteins by performing immunoprecipitation from U2OS cells. As shown in Figure 3a, BAP31 and STX17 were endogenously associated, but BAP31 and other proteins did not associate. In normal human fibroblast cells, BAP31 is also associated with STX17 (Figure S6), which suggests that BAP31 associates with STX17 in both normal cells and cancer cells. Next, we examined whether STX17 is involved in ER stress-induced autophagy. As shown in Figure 3b, *STX17* knockdown by siRNA silencing suppressed ER stress increased LC3-II expression, indicating that STX17 plays an important role in ER stress-induced autophagy. As shown in Figure 3c, co-immunoprecipitation assay revealed that BAP31 and STX17 were endogenously associated and BFA treatment decreased their interaction. To detect and visualize BAP31–STX17 interaction with or without ER stress, we performed confocal imaging analysis using the DuoLink method. First, we confirmed STX17 antibody specificity in immunofluorescence analysis, as examined by *STX17* knockdown and FLAG–STX17 overexpression. As shown in Figure S7a, STX17 antibody showed localized STX17 immunofluorescence activity in siControl-treated cells. However, *STX17* knockdown decreased STX17 immunofluorescence activity, and fluorescence diffused in all the cells in highly exposed images. In addition, FLAG–STX17-overexpressed U2OS cells were subjected to immunofluorescence analysis using FLAG and STX17 antibodies. These two antibodies showed strong fluorescence activity in FLAG-STX17-overexpressed U2OS cells (Figure S7b). For the immunofluorescence assay, we used STX17 antibody to detect STX17 in U2OS cells. Analysis using antibodies recognizing BAP31 [12] and STX17 showed that BAP31 appears to co-localize with STX17 (yellow), and Spearman correlation showed that this co-localization significantly decreases ER stress response (Figure 3d,left,e). Using the DuoLink method, we found positive signals indicating BAP31-STX17 interactions; these signals decreased under ER stress (Figure 3d, right). As shown Figure S7c, most of the DuoLink signals of BAP31–STX17 interaction were on the ER and in close vicinity to mitochondria, indicating that BAP31–STX17 interaction exists in ER–mitochondria contact sites and other ER areas. These results suggested that BAP31 is endogenously associated with STX17 in ER–mitochondria contact sites and other ER areas and that this interaction collapses under ER stress.

It was previously reported that autophagosome formation requires binding of STX17 to ATG14L. ATG14L is a unique subunit that interacts with the autophagy-specific phosphatidylinositol-3-OH kinase (PI(3)K) complex [20]. Therefore, we hypothesized that BAP31 suppresses STX17 and ATG14L association by interacting with STX17. We investigated whether the BAP31 suppresses STX17 and ATG14L interaction using HA-tagged ATG14L expression in U2OS cells. The co-immunoprecipitation experiments demonstrated that STX17 did not interact with HA-ATG14L in normal conditions, but STX17 associated with HA-ATG14L in *BAP31* knockdown, ER stress and starvation conditions (Figure 3f and Figure S8). We confirmed the endogenous interactions between STX17 and ATG14L by confocal imaging analysis with the DuoLink method. As shown Figure 3g (left and right panels), STX17 and ATG14L interaction signals were detected in *BAP31* knockdown and/or ER stress condition and BAP31 knockdown stimulated STX17 and ATG14L interactions, compared to ER stress conditions. These results suggest that BAP31 has a role in the inhibition of autophagy induction via partial suppression of STX17 and ATG14L interactions.

### 3.4. Loss of BAP31 Stimulates Tumor Invasion and Growth In Vitro and In Vivo

**Figure 4:**
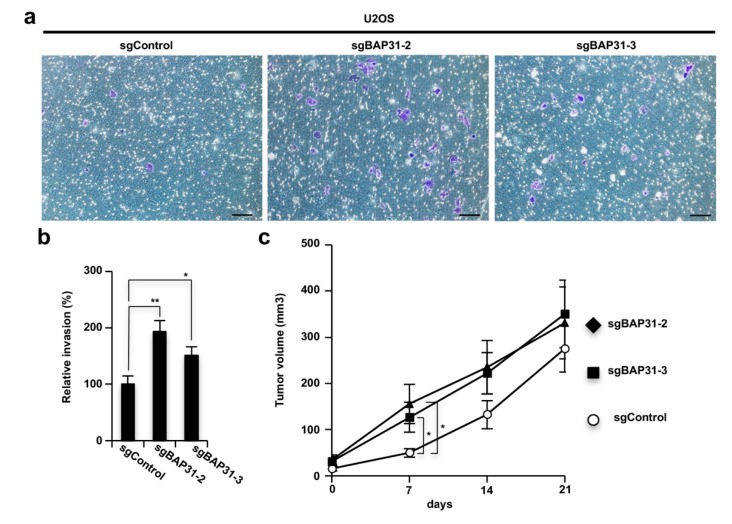
BAP31 deficiency leads to stimulation of tumor invasion and growth in vitro and in vivo. (**a**,**b**) BAP31 deficiency stimulates invasion activity in U2OS cells. U2OS cells subjected to *BAP31* knockout via the CRISPR-Cas9 system (sgControl, sgBAP31-2, and sgBAP31-3) were seeded onto a transwell chamber coated with Matrigel. After 24 h, invader cells were stained with crystal violet (scale bar, 100 µm) (a). Stained crystal violet was extracted and measured at 570 nm (b). Data are presented as the mean ± SD of the three simultaneously performed experiments (**b**). (**c**) The knockout of *BAP31* stimulates tumor growth at early time points in nude mice. U2OS sgControl, sgBAP31-2, or sgBAP31-3 cells were used for xenograft transplantation in nude mice. After four days, the tumor volumes were measured on the indicated days. The values represent the mean ± standard error of the eight mice from each group. The *P*-value was calculated using two-way ANOVA. n.s., not significant; * *P* < 0.05; ** *P* < 0.01 (**b**,**c**).

The induction of autophagy is important to cell adaptation to environments with limited nutrition, as seen when cancer cells develop and stimulate invasion activity and increase the growth of xenografted tumors [21]. Therefore, we confirmed that BAP31 knockout cells exhibit enhanced tumorigenicity, consistent with the phenotype of highly-induced autophagy. We examined cell invasion activity by performing a Boyden-chamber assay using a Matrigel coated chamber and tumor growth in vivo using a tumor xenograft model. The *BAP31* knockout U2OS cells (sgBAP31-2 and sgBAP31-3) showed increased invasion activity compared to the control cells (sgControl) (Figure 4a,b), and the tumors induced by the *BAP31* knockout U2OS cells grew more aggressively than those induced by the control cells at the early time point (7 days) (Figure 4c). Therefore, the loss of BAP31 stimulated tumor activity, indicating that BAP31 has a homeostatic role in suppressing tumor cell adaptation in the initiation of cancer cell development.

## 4. Discussion

This study demonstrated that BAP31 is a key factor in negatively regulated autophagy induction and positively regulated ER stress-induced cell death, which inhibits cell adaptation to ER stress conditions. By performing a co-immunoprecipitation assay, we identified STX17 as a protein associated with BAP31. The BAP31-STX17 complex is critical for the inhibition of STX17 and ATG14L interaction on the induction of autophagy signaling. Furthermore, the loss of BAP31 stimulated cellular invasion activity and tumor growth in vivo.

Previous studies have indicated that BAP31 is involved in apoptotic signaling to pro-apoptotic molecules of the Bcl-2 family, such as Bax and Bak, which lead to mitochondrial cytochrome c release and the transmission of apoptotic signals from the ER to the mitochondria via a physical interaction between BAP31 and mitochondria-localized Fis1 or Bcl-2 [11,13,22]. However, the function of BAP31 in the signaling for autophagy induction is unclear. Here we provide new insight into the suppressive role of BAP31 in the regulation of autophagy signaling via association with STX17. STX17 is a localized ER membrane protein that interacts with ATG14L in the formation of functional autophagosomes [23]. STX17 also regulates mitophagy by interacting with Fis1. Collapse of this interaction induces aberrant STX17 accumulation on mitochondria and then triggers mitophagy induction [24]. BAP31 also interacts with Fis1 and STX17, indicating that BAP31 might be involved in this mitophagy-regulated pathway. Therefore, BAP31 has a dual role, stimulating cell death upon ER stress and antagonizing autophagy induction.

An important point of the present study is the demonstration that *BAP31* knockdown suppressed ER stress-induced cell death and *BAP31* knockout human osteosarcoma cells stimulated the growth of tumors in nude mice, suggesting that loss of BAP31 may enhance cancer cell adaptation to low nutrient conditions as known ER stress. The initiation from a normal cell to a cancer cell requires a mutation in the genome that eliminates growth restriction, but at the same time, it must be adapted to grow in an environment of nutrient deficiency [1,2,3]. This is known as a metabolic checkpoint and overcoming this is necessary for the progression of cancer cells, suggesting that BAP31 inhibits the ability of the cells to overcome the metabolic checkpoint.

Recent studies have reported that silencing BAP31 suppresses tumor proliferation in cervical cancer cell lines and that BAP31 is important for virus infection and the proliferation of human papilloma virus–infected cells [25,26,27,28]. Other reports indicated that miR-451a has anti-tumor effects to target BAP31 in colorectal cancer cell lines [29]. Studies have also reported that miR-451a has antitumor effects targeting BAP31 in colorectal cancer cell lines. In this study, we used three types of cells, the osteosarcoma cell line U2OS, the cervical cancer cell line HeLa, and normal MEF cells, which showed that *BAP31* knockdown significantly suppresses ER stress-induced apoptosis and treatment with siBAP31 alone slightly decreases viability and induces apoptosis. However, these effects were not significant in our experimental conditions. We also determined that *BAP31* knockdown induces autophagy in these three cell lines [12]. However, we investigated tumor invasion and growth assay in only U2OS cells. Therefore, BAP31 is a key factor for ER stress-induced apoptosis, and BAP31 suppression induces autophagy. However, BAP31 might play a different role in tumor proliferation in vivo, depending on the cell type and cell line. Analysis of available sequencing data (http://www.cbioportal.org/public-portal/index.do) of patient samples with several types of cancers points to the presence of genomic alterations in *BAP31* in subsets of patients with lymphoma (10.4%) and esophageal cancers (5.0%), in addition to patients with epithelial cancers (e.g., gastric, bladder and ovarian cancers). The overall findings demonstrated a novel function for BAP31 as a tumor suppressor–like factor that acts through two prominent regulatory routes: ER stress-induced apoptosis and autophagy suppression via BAP31–STX17 interaction.

## Supplementary Materials

The following are available online at https://www.mdpi.com/2073-4409/8/11/1350/s1, Figure S1: The quantification analysis for Figure 1a,b. Figure S2: ER stress induced autophagy and cell death is suppressed by knockdown of ATG7. Figure S3: The overexpression of BAP31 suppresses ER stress-induced autophagy. Figure S4: The suppression of BAP31 reduces p62 expression levels. Figure S5: The loss of BAP31-suppressed ER stress-induced apoptosis by inducing autophagy. Figure S6: BAP31 interacts with STX17 in human normal fibroblast cells. Figure S7: BAP31 interacts with STX17 on the ER and ER-mitochondria contact sites in U2OS cells. Figure S8: *BAP31* knockdown stimulates starvation induced STX17 and ATG14 interaction in U2OS cells.
