# Supplementary material for: BAP31 Inhibits Cell Adaptation to ER Stress Conditions, Negatively Regulating Autophagy Induction by Interaction with STX17
Source: Cells. 2019 Oct 30;8(11):1350. doi: 10.3390/cells8111350 (PMC6912744; doi:10.3390/cells8111350)
Supplement: Supplementary file 1 [file cells-08-01350-s001.pdf]

Supplementary Materials for

BAP31 Inhibits Cell Adaptation to ER Stress Conditions, Negatively Regulating Autophagy Induction by Interaction with STX17

Kayo Machihara [1,2,†] and Takushi Namba [1,3,†,*]

[1] Research and Education Faculty, Multidisciplinary Science Cluster, Interdisciplinary Science Unit, Kochi University, Kohasu Oko-cho Nankoku-shi, Kochi, 783-8505, Japan
[2] Graduate School of Medicine, Kochi University, Nankoku 783-8502, Japan
[3] Department of Marine Resource Science, Faculty of Agriculture and Marine Science, Kochi University, Nankoku 783-8502, Japan

* Correspondence: t-namba@kochi-u.ac.jp; Tel.: +81-88-880-2184
† These authors contributed equally to this work.

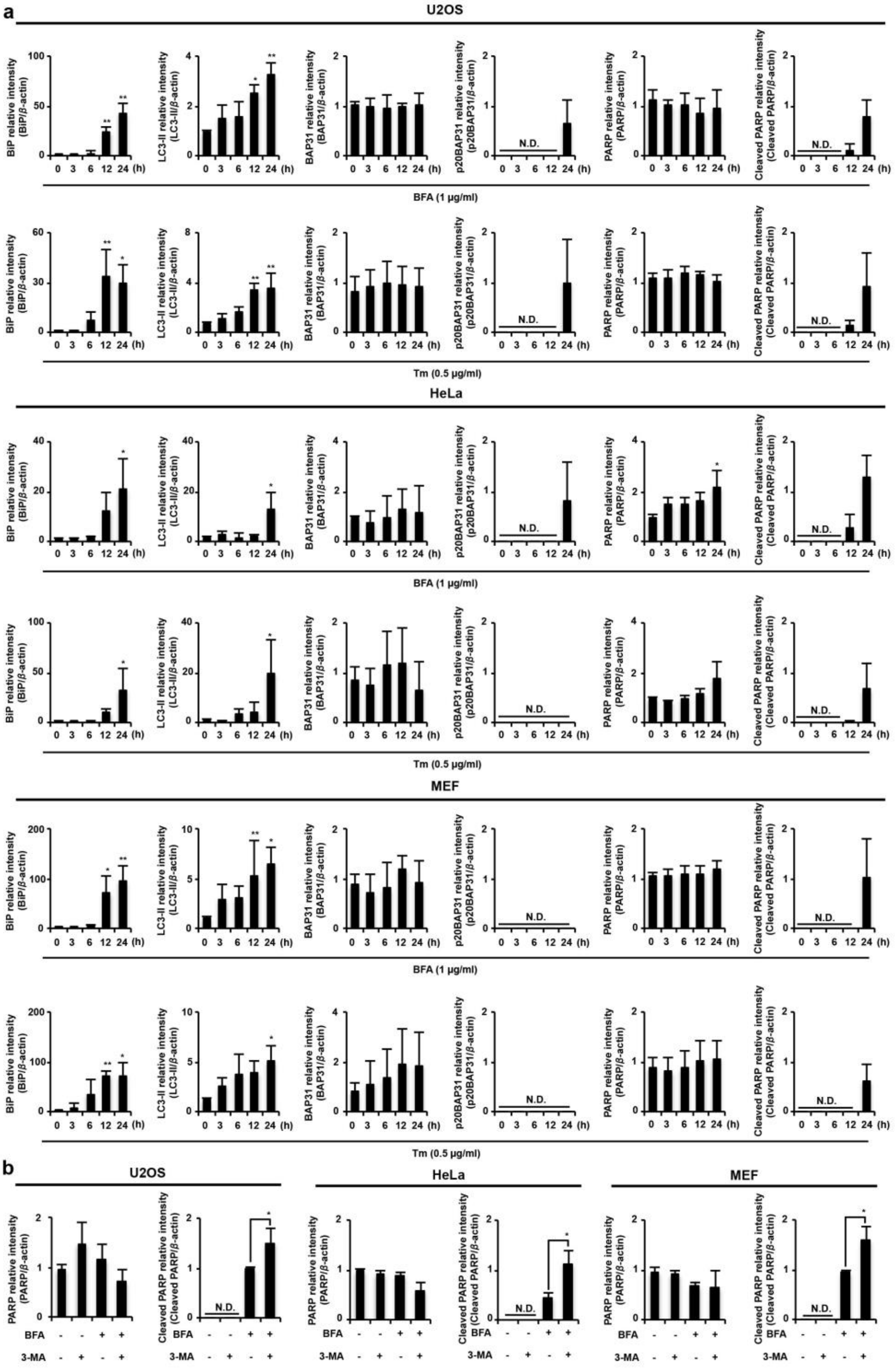

Supplementary Figure S1. The quantification analysis for Figure 1a,b.

Values were normalized to β-actin gene expression and expressed relative to the control sample (i.e. 0 h (BiP, LC3-II, BAP31 and PARP) or 24 h (p20BAP31 and cleaved PARP)). Values are given as mean ± S.D. (n=3). $^{**}P < 0.01$; $^{*}P < 0.05$ (a, b). Not detectable (N.D.)

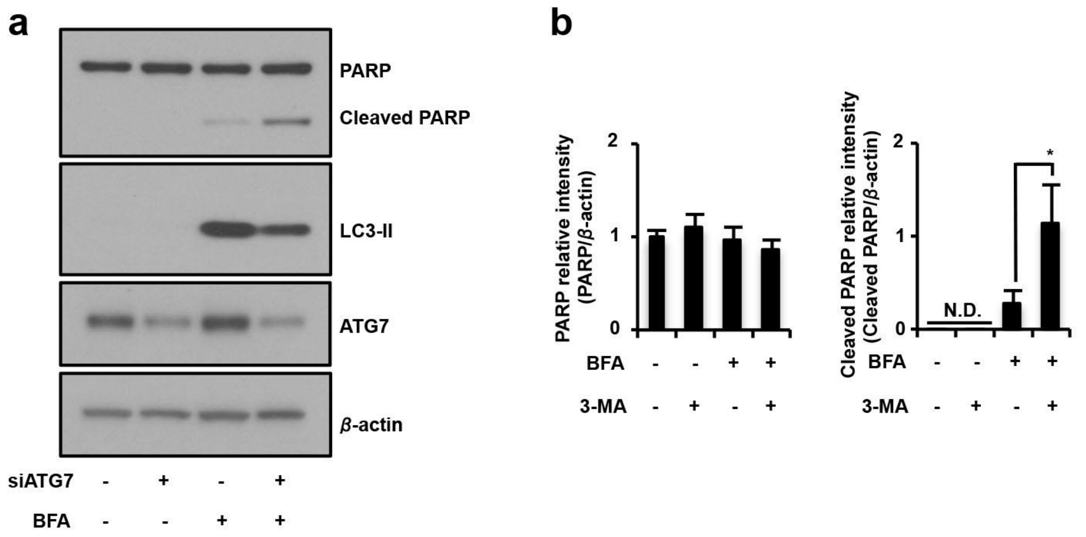

Supplementary Figure S2. ER stress induced autophagy and cell death is suppressed by knockdown of ATG7.

(**a**) The cells were transfected with siATG7 (+) or siControl (−) for 16 h and these cells were incubated with or without BFA (1 μg/mL) for 18 h in U2OS cells. Cells were subjected to immunoblotting using indicated antibodies. (**b**) Values were normalized to β-actin gene expression and expressed relative to the control sample (i.e. 0 h (PARP) or 24 h (cleaved PARP)). Values are given as mean ± S.D. (n = 3). *$P < 0.05$ (b). Not detectable (N.D.).

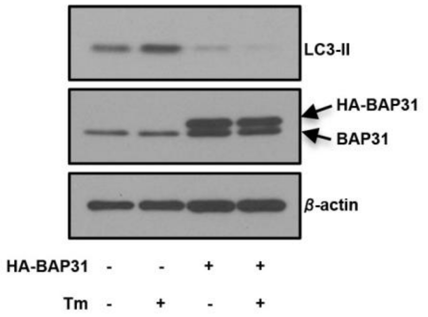

Supplementary Figure S3. The overexpression of BAP31 suppresses ER stress-induced autophagy.

The cells were transfected with HA-BAP31 (+) or pcDNA3.1 (−) empty vector for 16 h and these cells were incubated with or without 0.5 μg/mL of Tm for 18 hour. Cells were subjected to immunoblotting using indicated antibodies. This experiment was repeated two times.

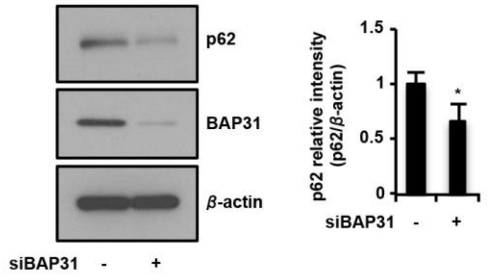

Supplementary Figure S4. The suppression of BAP31 reduces p62 expression levels.

U2OS cells were transfected with 150 pmol of siBAP31 or siControl for 24 h. Cells were subjected to immunoblotting using indicated antibodies. Values were normalized to β-actin gene expression and expressed relative to the control sample (i.e. siControl (−)). Values are given as mean ± S.D. (n = 3). *$P <$ 0.05

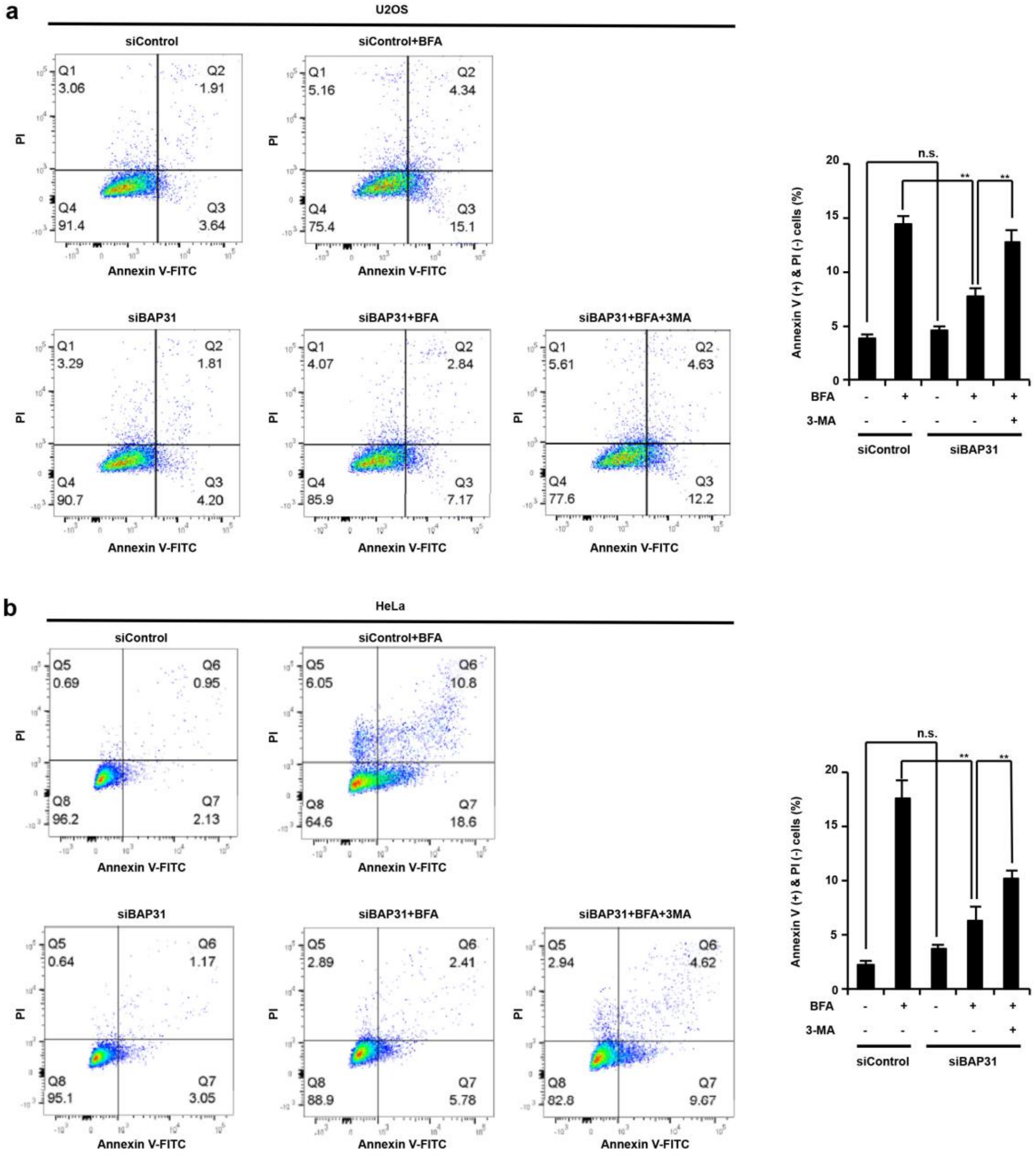

Supplementary Figure S5. The loss of BAP31-suppressed ER stress-induced apoptosis by inducing autophagy.

(**a**, **b**) The cells were transfected with siBAP31 or siControl for 16 h and these cells were preincubated with or without 5 mM of 3-MA for 1 hour and further incubated with or without BFA (1 μg/mL) for 10 h in U2OS cells (**a**) or 15 h in HeLa cells (**b**). The apoptotic cell numbers were determined by FACS (Annexin V-FITC and PI double staining). Values are given as mean ± S.D. (n = 3). $^{**}P < 0.01$; n.s. , not significant (**a**, **b**).

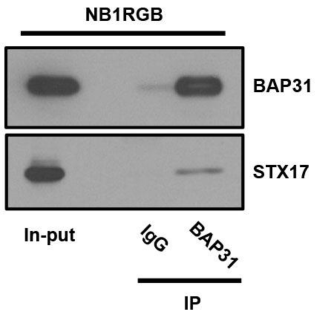

Supplementary Figure S6. BAP31 interacts with STX17 in human normal fibroblast cells.

U2OS cells were harvested, and the proteins were cross-linked with DSP prior to the protein extraction. A coimmunoprecipitation assay was performed with cell lysates, followed by western blotting, using the indicated antibody. This experiment was repeated two times.

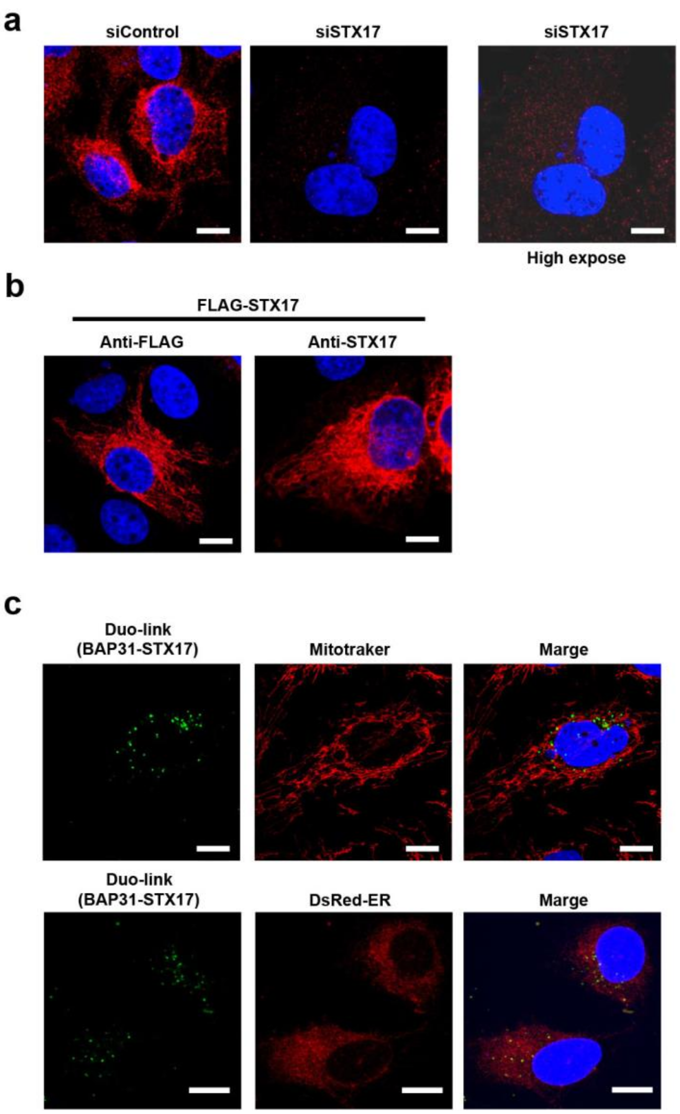

Supplementary Figure S7. BAP31 interacts with STX17 on the ER and ER-mitochondria contact sites in U2OS cells.

(**a**, **b**) BAP31 knockdown (**a**) or FLAG-STX17 expression (**b**) U2OS cells were subjected to immunostaining analysis using antibodies to STX17 (red) or FLAG (red). (**c**) Endogenous BAP31 and STX17 interactions were detected by performing a PLA and are shown as green dots. Red represents mitochondria MitoTracker staining (upper panel). Red represents ER DsRed-ER expression (Lower panel). Merged images are also shown, and colocalization is indicated in yellow. Blue represents nuclear DAPI staining (scale bar, 10 μm).

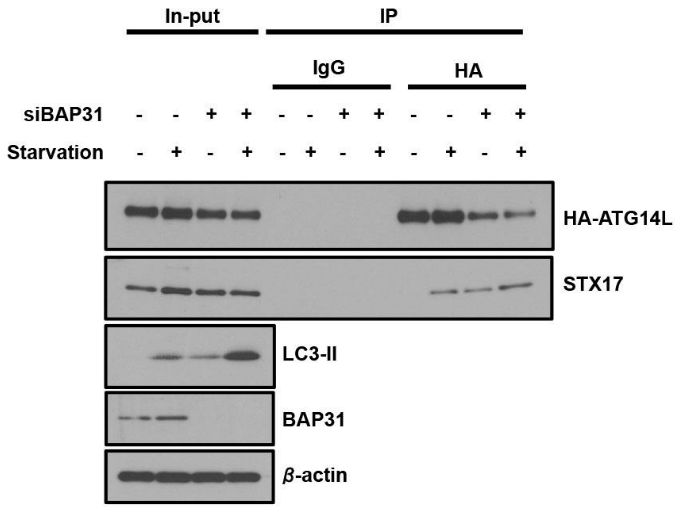

Supplementary Figure S8. *BAP31* knockdown stimulates starvation induced STX17 and ATG14 interaction in U2OS cells.

U2OS cells were transfected with (+) or without (−) HA-ATG14 and siControl(−) or siBAP31(+) for 24 h and these cells were then treated with (−) or without (+) stavation for 4 h. A coimmunoprecipitation assay was performed with cell lysates, followed by western blotting, using the indicated antibodies. This experiment was repeated two times.
